# A recommendation to perform a blood culture before the administration of intravenous antibiotics increased the detection of *Staphylococcus aureus* bacteremia

**DOI:** 10.1007/s10096-013-2013-7

**Published:** 2013-11-19

**Authors:** A. Jogenfors, L. Stark, J. Svefors, S. Löfgren, B.-E. Malmvall, A. Matussek

**Affiliations:** 1 Faculty of Health Sciences, Linköping University, Linköping, Sweden; 2 Department of Laboratory Services, Division of Medical Services, County Hospital Ryhov, Jönköping, Sweden; 3 Department of Infectious Diseases, County Hospital Ryhov, Jönköping, Sweden; 4 Futurum—The Academy for Healthcare, Jönköping County Council, Jönköping, Sweden

## Abstract

In 2004, the Surviving Sepsis Campaign was launched to increase awareness and improve the outcome of severe sepsis. Accordingly, in Jönköping County, Sweden, a strong recommendation to perform a blood culture before the start of intravenous antibiotic treatment was introduced in 2007. Moreover, a reminder was included in the laboratory report to consult an infectious disease specialist when *Staphylococcus aureus* was isolated from a blood culture. Retrospectively, patients with at least one blood culture growing *S. aureus* during 2002 through 2003 (pre intervention *n* = 58) or during 2008 through 2009 (post intervention *n* = 100) were included. Medical records were evaluated regarding clinical data and outcome. Blood culture isolates were characterized by antibiotic susceptibility testing (AST) and *S. aureus* protein A (*spa*) gene typing. The annual incidence of *S. aureus* bacteremia (SAB) increased from 28 per 100,000 inhabitants at the pre intervention period to 45 per 100,000 at the post intervention period (*p* = 0.046). During post intervention, the SAB incidence was significantly higher in men (*p* = 0.009). The mortality rate during hospital stay was 14 % during pre intervention and 18 % during post intervention (*p* = 0.47). The most common *spa* types were t012 and t084. The Surviving Sepsis Campaign resulted in an increased number of detected cases of SAB. The mortality rate was the same before and after the intervention, and no *spa* type correlated to certain clinical manifestations or mortality.

## Background

*Staphylococcus aureus* bacteremia (SAB) correlates to sepsis, severe sepsis, and septic shock with high mortality [1]. An early diagnosis and a structured management and treatment of patients with SAB are essential to prevent a fatal progress [2, 3]. Guidelines for the management of severe sepsis and septic shock were introduced in 2004 [4]. The rate of methicillin-resistant *S. aureus* (MRSA) in Sweden is low [5]; however, knowledge of the local prevalence of MRSA is crucial in the choice of empirical treatment [6].

In Jönköping County in the southern part of Sweden, a patient safety project aiming at the early identification and treatment of patients with suspected severe sepsis was introduced in 2007, in accordance with Dellinger et al. [4]. This included a strong recommendation to perform a blood culture before the start of an intravenous antibiotic treatment and a reminder in the laboratory report to consult an infectious disease specialist when *S. aureus* was isolated from a blood culture.

In this study, we investigate the incidence and outcome of SAB in Jönköping, Sweden, 2002 through 2003 and 2008 through 2009 to evaluate if the patient safety project resulted in an increase in SAB cases detected. Furthermore, we investigate the molecular epidemiology of *S. aureus* based on sequencing of the repeat region of the *S. aureus* protein A gene (*spa* typing) [7].

## Methods

### Setting

The study was performed at the County Hospital Ryhov in Jönköping, situated in the southern part of Sweden. The hospital has 638 beds and 3,500 employees. Inhabitants of the catchment area with an age above 18 years averaged 105,300 in the pre intervention period and 112,900 in the post intervention period.

### Study design

This retrospective study is based on the evaluation of medical records from all patients (*n* = 158) with SAB at a pre intervention period of 2 years (2002 and 2003) (*n* = 58) and a post intervention period of 2 years (2008 and 2009) (*n* = 100) comparing medical history, clinical findings, and laboratory results. No independent evaluation of the clinical diagnosis was performed. For epidemiological purposes, *spa* typing of *S. aureus* in blood culture isolates was performed. In total, 163 *S. aureus* isolates from 148 different patients were available for *spa* typing. To evaluate the long-term effect of the intervention, figures (number of blood cultures, number of patients with SAB, and annual incidence) from the 2 years 2011 and 2012 were included as a follow up.

### Intervention

In 2004, new guidelines, aiming at an increased attention to patients at risk for severe sepsis, were introduced. The guidelines included a strong recommendation to always perform a blood culture before administrating the first intravenous antibiotic dose and a reminder in the laboratory report to consult an infectious disease specialist when *S. aureus* was isolated from a blood culture. This was implemented in Jönköping between 2003 and 2008, and, noteworthy, the nurse was committed to be responsible for taking a blood culture before starting the antibiotic treatment.

### Definitions

SAB was defined as *S. aureus* obtained from one or both blood culture bottles. Severe sepsis was defined according to the American College of Chest Physicians/Society of Critical Care Medicine Consensus Conference Committee [8]. Nosocomial infections were defined as a positive blood culture taken later than 48 h after arrival to the hospital. The mortality was the all-cause mortality. Recurrence was defined as a new episode of SAB within 1 year, but more than 4 weeks after the time of the first diagnosis. A relapse was defined as a recurrence caused by the same *spa* type as the primary episode and a reinfection was defined as a recurrence with a different *spa* type.

### Antibiotic susceptibility testing

Antibiotic susceptibility testing (AST) was performed by the disk diffusion method on Iso-Sensitest Agar (Oxoid, Ltd., Basingstoke, UK) using specific antibiotic disks (Oxoid, Ltd., Basingstoke, UK) pre and post intervention according to the Swedish standard for AST (http://www.srga.org). The recommendations and antimicrobial susceptibility testing criteria of the Swedish Reference Group of Antibiotics (SRGA) were adhered to. The SRGA has, since 2002, adopted all recommendations on breakpoints and methodology from the European Committee on Antimicrobial Susceptibility Testing (EUCAST).

### *spa* typing

The *spa* typing of blood culture isolates (stored in skimmed milk at −80 °C) was performed as previously described [7, 9]. Sequencing was performed by GATC Biotech (GATC Biotech AG, Konstanz, Germany) and each isolate was assigned an *spa* type using the Ridom StaphType software (v1.5.21, Ridom GmbH, Würzburg, Germany). The Ridom StaphType software was also used to obtain based upon repeat pattern (BURP) clusters [10], enabling the determination of clonal relatedness. The default parameters (*x* = 5; *y* = 4) were applied.

### Panton–Valentine leukocidin testing

Panton–Valentine leukocidin (PVL) testing was performed on blood culture isolates from patients with pneumonia, according to Lina et al. [11].

### Statistical analyses

IBM SPSS Statistics 19 and Microsoft Excel 2007 were used. A two-sided Chi-square test with one degree of freedom was used for the evaluation of proportions and the Mann–Whitney test was used for calculations of differences between means. A *p*-value ≤0.05 was considered significant. Antibiotic-sensitive and -intermediate isolates were combined in statistical analyses.

### Ethical considerations

This study complied with current Swedish laws.

## Results

The number of blood cultures increased from 8,616 at pre intervention to 14,842 at post intervention (72 %, *p* < 0.0001). The number of patients suffering from SAB during the pre intervention period was 58 and at post intervention it was 100 (73 %, *p* = 0.0037). The proportion of patients with SAB was the same during the pre and post intervention periods. The annual incidence of SAB increased from 28 per 100,000 inhabitants at pre intervention to 45 per 100,000 at post intervention (*p* = 0.046) (Table 1). At pre intervention, no statistically significant difference (*p* = 0.071) in the SAB rate was seen between women and men, whereas at post intervention, the SAB rate was significantly higher in men (*p* = 0.009). The number of blood cultures performed was significantly higher for men compared to women at pre (*p* < 0.0001) and post (*p* < 0.0001) intervention (Table 1), and the increase of performed blood cultures was higher for men than for women (*p* < 0.0001).

**Table 1:** Annual incidence of *Staphylococcus aureus* bacteremia (SAB)

	2002–2003	*p*-Value^a^	2008–2009	*p*-Value^a^	%^b^
Blood cultures	8,616		14,842	<0.0001^c^	+72
Men	4,538		8,224		+81
Women	4,078	<0.0001^d^	6,618	<0.0001^d^	+62
Number of positive blood cultures with *S. aureus*	161		218	0.019^c^	+35
SAB	58		100	0.0037^c^	+72
Men	35		62		+77
Women	23	0.071^d^	38	0.009^d^	+65
Annual incidence per 100,000	28		45	0.046^c^	+61

^a^Significant if *p*-value ≤ 0.05

^b^Difference between pre and post intervention

^c^Compares the years 2002–2003 with 2008–2009

^d^Compares men and women for each period of time

**Fig. 1:**
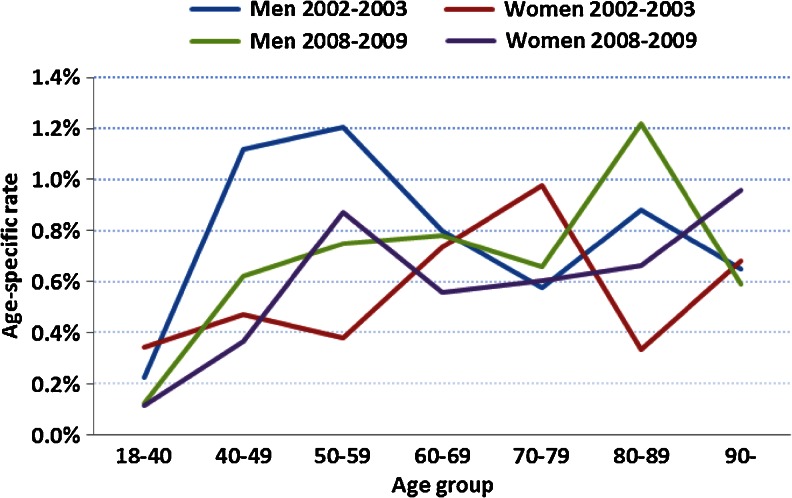
Age-specific rates of cases with *Staphylococcus aureus* bacteremia (SAB) during the years 2002–2003 and 2008–2009

The median age at SAB diagnosis was 69 and 75 years for men (*p* = 0.11) and 74 and 74 years for women (*p* = 0.33) during pre and post intervention, respectively. For men, the age-specific SAB rates were highest at 50–59 years of age at pre intervention and 80–89 years at post intervention. For women, the corresponding figures were 70–79 years at pre intervention and 50–59 years at post intervention (Fig. 1). At follow up, a further increase (*p* < 0.0001) in the number (17,783) of blood cultures performed was noted, as well as a sustained rate (*p* = 0.35) of patients with SAB (*n* = 115) and annual incidence of 44 per 100,000 (*p* = 0.92).

The mortality among SAB patients during hospital stay was 14 and 18 % at pre and post intervention, respectively. The 12 months mortality was 50 and 44 % at pre and post intervention, respectively (Table 2). There was no difference in the mortality between men and women.

**Table 2:** All-cause mortality after *Staphylococcus aureus* bacteremia (SAB) and clinical manifestations at pre and post intervention

	Years 2002–2003 (%)		*p*-Value^a^	Years 2008–2009 (%)		*p*-Value^a^
	Men	Women		Men	Women	
Mortality						
During hospital stay	6 (17.1)	2 (8.7)	0.36	11 (17.7)	7 (18.4)	0.93
Within 3 months after discharge	5 (14.3)	4 (17.4)	0.75	8 (12.9)	4 (10.5)	0.72
During 3–12 months after discharge	9 (25.7)	3 (13.0)	0.24	11 (17.7)	3 (7.9)	0.17
Total	20 (57.1)	9 (39.1)	0.18	30 (48.3)	14 (36.8)	0.25
Clinical manifestations^b^	Total			Total		
Skin or soft tissue injury	8 (14)			29 (29)		0.03
Endocarditis	7 (12)			9 (9)		0.54
Spondylitis	4 (7)			11 (11)		0.4
Osteitis	4 (7)			6 (6)		0.82
Arthritis	3 (5)			7 (6)		0.65
Prosthetic infection	1 (2)			2 (2)		0.90
Pneumonia	1 (2)			16 (16)		0.0052
Unclear focus	23 (40)			25 (25)		0.054

^a^Significant if *p*-value ≤ 0.05

^b^Manifestations may occur more than once in an individual

Endocarditis, spondylitis, and pneumonia were the most common manifestations related to SAB. During the pre intervention period, none of the seven patients with endocarditis was sent to cardiac surgery during hospital stay; however, four out of nine patients were sent to cardiac surgery at post intervention. A major part of patients with SAB had an unclear focus (Table 2).

The number of patients suffering from a nosocomial disease was 12 (21 %) at pre intervention and 24 (24 %) at post intervention (*p* = 0.63). The time from arrival at the hospital to the first antibiotic dosage administered (not including nosocomial disease) was, on average, 14 h (median 6 h) at pre intervention and 11 h (median 4 h) at post intervention (*p* = 0.38). The mean duration of intravenous antibiotic treatment was 13 days at pre intervention and 15.5 days at post intervention (*p* = 0.23). Cefuroxime was the most common antibiotic drug at the start of treatment pre intervention, given to 45 (78 %) patients, and cefotaxime at post intervention, given to 25 patients (25 %). Cloxacillin was given to 79 patients (50 %) during treatment, but was used as the first antibiotic in only 12 cases (8 %). Oral antibiotics after a period of intravenous antibiotics were given to 48 patients (83 %) pre intervention and to 67 patients (67 %) post intervention (*p* = 0.03).

At pre intervention, 45 out of 58 patients (78 %) and at post intervention, 69 out of 100 patients (69 %) were referred to a specialist in infectious diseases (*p* = 0.25), and 19 (33 %) and 26 (26 %) at pre and post intervention, respectively, were treated at the infectious disease department (*p* = 0.36). Hospitalization was delayed (median 1 day) for 28 and 39 % (*p* = 0.15) of patients at pre and post intervention, respectively. The mortality rate of these patients did not differ from the mortality of patients immediately hospitalized at the first medical contact (*p* = 0.35). The number of SAB patients treated at the intensive care unit increased from four (7 %) patients pre intervention to 22 (22 %) patients post intervention (*p* = 0.02). Heart ultrasound examination was performed on 30 patients at pre intervention (52 %) and on 76 patients at post intervention (76 %) (*p* = 0.0017). The average length of hospital stay was 20 days at pre intervention and 24 days at post intervention (*p* = 0.81).

### *spa* typing

**Fig. 2:**
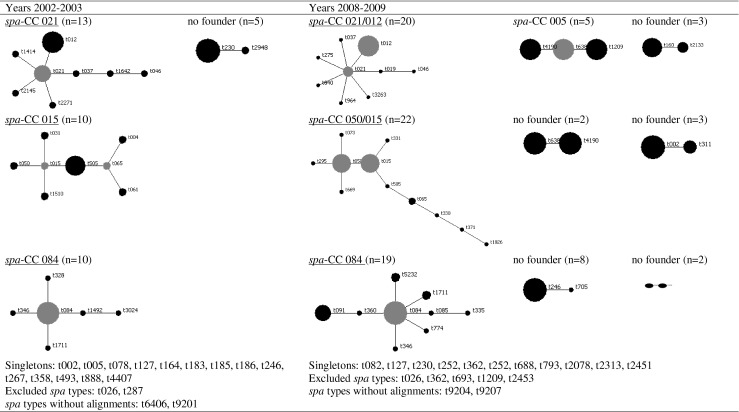
Based upon repeat pattern (BURP) clustering of 60 isolates from years 2002–2003 and 103 isolates from years 2008–2009 based on *spa* types. The *spa* type with the highest founder score is defined as the founder of the cluster and is given the cluster its name. Subfounders are the *spa* types with the second highest founder score. Founders and subfounders are marked in *gray*. Each *dot* in a cluster represents a unique *spa* type and the diameter of the dot represents the quantity of the *spa* type

In total, 163 out of 173 isolates were available for *spa* typing from 148 of the 158 patients. During pre and post intervention, 42 and 58 different *spa* types were detected, respectively. The BURP cluster analysis resulted in three dominating *spa* clonal complexes (CCs) (Fig. 2). These comprised 55 and 61 % of all *spa* types at pre and post intervention, respectively. The three clusters were equally distributed within each time period and between the two periods. Minor differences in the *spa* type distribution between pre and post intervention were noted (Fig. 2).

A total of 12 patients suffered from 13 recurrences. All isolates were available for *spa* typing from both the primary infection and the recurrence. Eleven patients had relapses and two patients had a reinfection. The *spa* types seen in the reinfected patients were t9201/t246 and t183/t505, respectively. Two patients had a dual infection, i.e., *S. aureus* of different *spa* types isolated from the same bottle. These *spa* types were t021/t037 (resistant to fusidic acid) and t084/t362, respectively. No *spa* type was associated with increased risk of death or clinical manifestations (Table 2).

### AST

All of the 171 out of 173 *S. aureus* isolates analyzed were sensitive to clindamycin, tobramycin, trimethoprim–sulfamethoxazole, vancomycin, erythromycin, rifampicin, netilmicin, linezolid, quinolones meropenem, cefadroxil, and cefuroxime. Fusidic acid AST resulted in 14 resistant isolates pre intervention compared to one resistant isolate post intervention. No MRSA isolates were detected.

### PVL testing

No PVL-positive isolates were found.

## Discussion

The main finding of this study is that the implementation of a quality improvement program, aiming at the early detection of patients with severe sepsis, resulted in a 61 % increase in the annual incidence of SAB, which was sustained at the 3 years follow up. This indicates that a substantial number of SABs remained undetected before the intervention. The greatest increase in SAB was seen in men, resulting in a significantly higher SAB rate in men than in women at post intervention, which is in agreement with other reports [12, 13]. Furthermore, no *spa* types correlated to increased risk of death or specific clinical manifestations.

Our pre intervention SAB incidence figures are in line with the results of Jacobsson et al. [14] performed in geographic proximity and in a multinational study [5], whereas our post intervention figures are significantly higher. Both studies included, in contrast to our study, patients below 18 years of age. The retrospective design of our study may have underestimated the true incidence [15]; however, our increased sampling may have compensated for this and, thus, better reflects the true SAB incidence in the population.

The mortality rates during hospital stay and after 12 months did not change significantly between pre and post intervention. Hence, the SAB patients detected through the quality improvement program seems to have comparable mortality as the pre intervention patients. In contrast to a recent report by Jacobson and Nasic showing increased mortality in women, we show similar mortality rates between men and women [16].

The numbers of clinical manifestations are too few to draw significant conclusions. However, one serious and common manifestation was infective endocarditis [17], seen in approximately 10 % of the patients in this study. Notably, although we observed an increase in transesophageal or transthoracic echocardiography between pre and post intervention, the rate of endocarditis remained stable. None of the patients with endocarditis were sent to cardiac surgery during pre intervention compared to four out of nine patients during post intervention, which may reflect modified treatment recommendations.

Among the other clinical manifestations, pneumonias and skin and soft tissue infections were higher at post intervention. Only the isolates from the patients suffering from pneumonia were PVL tested and none were positive. The relatively high proportion of pneumonias should, therefore, be interpreted with caution, since the study was performed retrospectively without an independent evaluation of the clinical diagnosis.

Between pre and post intervention, we found an average non-significant decrease of 3 h from admission to hospital to the start of antibiotic treatment and longer duration of intravenous treatment with antibiotics.

Although the intervention program included a recommendation to contact an infectious disease specialist, no increase in referrals was noted. Moreover, delay to hospitalization was not decreased.

We found a large diversity of *spa* types and stability over time for common types, which confirm recent Norwegian findings [18]. The common *spa* types were also found in recent reports on healthy individuals from the same geographic area [19–21], as in our study (Table 3). This is in agreement with the finding that a successful colonizing strain can transform into a life-threatening human pathogen at any time [22–24]. Notably, *spa* type t084, frequently found during both pre and post intervention, could not be detected in the study by Matussek et al. [19] performed during the time for our pre intervention, but was later detected in healthy individuals [20]. This may indicate that this type was first established in clinical infections in our region. No specific *spa* type correlated to clinical manifestations or mortality, although a correlation to mortality was recently reported [18].

**Table 3:** The most common isolates from other studies in comparison with the 14 most common *spa* types in this study

*spa* types	This study, *n* = 163	Aamot et al. [18], *n* = 353	Sangvik et al. [26], *n* = 1,113	Matussek et al. [19], *n* = 160	Mernelius et al. [20], *n* = 1,635
	No.(%) of isolates	No. (%) of isolates	No. (%) of isolates	No.(%) of isolates	No. (%) of isolates
t012	13 (8.0 %)	14 (4.0 %)	94 (8.4 %)	20 (12.5 %)	65 (4.0 %)
t084	11 (6.7 %)	46 (13.0 %)	84 (7.6 %)		104 (6.4 %)
t246	8 (4.9 %)				
t021	7 (4.3 %)		42 (3.8 %)	10 (6.25 %)	42 (2.6 %)
t050	7 (4.3 %)				
t015	7 (4.3 %)		38 (3.4 %)	10 (6.25 %)	50 (3.1 %)
t230	6 (3.7 %)				
t091	4 (2.5 %)				
t505	4 (2.5 %)				
t065	3 (1.8 %)	15 (4.2 %)	55 (4.9 %)		
t026	3 (1.8 %)				
t1711	3 (1.8 %)				
t002	3 (1.8 %)	18 (5.1 %)	30 (2.7 %)		41 (2.5 %)
t005	3 (1.8 %)				

Relapse was more common than reinfection, and the two patients with reinfection had *spa* types of great difference. The two patients with dual infection, in contrast, had two closely related *spa* types, which may indicate genetic changes during infection.

A generally low rate of antibiotic resistance was observed and no MRSA was detected. The most distinguished resistance was to fusidic acid, noted in 14 isolates during pre intervention compared to one isolate during post intervention. This may reflect an outbreak of *S. aureus* fusidic acid resistance among Swedish children with bullous impetigo during the mid-1990s [25].

## Conclusions

The strong recommendation to perform a blood culture prior to intravenous antibiotics resulted in an increased detection of *Staphylococcus aureus* bacteremia (SAB), especially in men. Hence, the SAB patients detected through the quality improvement program seems to have comparable mortality as the pre intervention patients. Specific *spa* types could not be correlated to clinical manifestations or mortality.
